# Novel Insights in the Fecal Egg Count Reduction Test for Monitoring Drug Efficacy against Soil-Transmitted Helminths in Large-Scale Treatment Programs

**DOI:** 10.1371/journal.pntd.0001427

**Published:** 2011-12-13

**Authors:** Bruno Levecke, Niko Speybroeck, Robert J. Dobson, Jozef Vercruysse, Johannes Charlier

**Affiliations:** 1 Department of Virology, Parasitology and Immunology, Faculty of Veterinary Medicine, Ghent University, Merelbeke, Belgium; 2 Institute of Health and Society, Université Catholique de Louvain, Louvain, Belgium; 3 Division of Health Sciences, School of Veterinary and Biomedical Sciences, Murdoch University, Murdoch, Australia; Universidad San Francisco de Quito, Ecuador

## Abstract

**Background:**

The fecal egg count reduction test (FECRT) is recommended to monitor drug efficacy against soil-transmitted helminths (STHs) in public health. However, the impact of factors inherent to study design (sample size and detection limit of the fecal egg count (FEC) method) and host-parasite interactions (mean baseline FEC and aggregation of FEC across host population) on the reliability of FECRT is poorly understood.

**Methodology/Principal Findings:**

A simulation study was performed in which FECRT was assessed under varying conditions of the aforementioned factors. Classification trees were built to explore critical values for these factors required to obtain conclusive FECRT results. The outcome of this analysis was subsequently validated on five efficacy trials across Africa, Asia, and Latin America. Unsatisfactory (<85.0%) sensitivity and specificity results to detect reduced efficacy were found if sample sizes were small (<10) or if sample sizes were moderate (10–49) combined with highly aggregated FEC (k<0.25). FECRT remained inconclusive under any evaluated condition for drug efficacies ranging from 87.5% to 92.5% for a reduced-efficacy-threshold of 90% and from 92.5% to 97.5% for a threshold of 95%. The most discriminatory study design required 200 subjects independent of STH status (including subjects who are not excreting eggs). For this sample size, the detection limit of the FEC method and the level of aggregation of the FEC did not affect the interpretation of the FECRT. Only for a threshold of 90%, mean baseline FEC <150 eggs per gram of stool led to a reduced discriminatory power.

**Conclusions/Significance:**

This study confirms that the interpretation of FECRT is affected by a complex interplay of factors inherent to both study design and host-parasite interactions. The results also highlight that revision of the current World Health Organization guidelines to monitor drug efficacy is indicated. We, therefore, propose novel guidelines to support future monitoring programs.

## Author Summary

The reduction in number of eggs excreted in stools after drug administration is a primary parameter to monitor the efficacy of drugs against parasitic worms. Guidelines on how to perform such a fecal egg count reduction test (FECRT) are provided by the World Health Organization. However, it remains unclear to which extent these guidelines are cost-effective. We, therefore, performed a simulation study in which the FECRT was performed under varying conditions to determine the critical values for sample size, the detection limit of the fecal egg count (FEC) method, mean baseline FEC, and variation of FEC across host population that allow for conclusive FECRT results. The results revealed that a reliable monitoring system demands a sample size of 200 subjects and that in some cases FECRT results may be thwarted by low mean baseline FEC. For this sample size, the detection of the FEC method or the variation of FEC across the host population did not affect the FECRT results. Our findings underscore that the current guidelines are not cost-effective, demanding too much financial and technical resources. We, therefore, propose novel guidelines to support future monitoring programs.

## Introduction

Infections with the soil-transmitted helminths (STHs), namely *Ascaris lumbricoides*, *Trichuris trichiura* and hookworm (*Necator americanus* and *Ancylostoma duodenale*) are among the most common infectious diseases in children of tropical countries causing malnutrition, growth stunting, intellectual retardation, and cognitive deficits [1]. Currently, the large-scale administration of benzimidazole drugs (i.e., albendazole and mebendazole) is the most widely used method to control morbidity due to STH infections, and a scale-up of these large-scale treatment programs is underway in Africa, Asia, and Latin America (donation of 400 million tablets of albendazole by GlaxoSmithKline and 200 million tablets of mebendazole by Johnson & Johnson). Rather than aiming to achieve eradication, these control programs are focused on reducing infection intensity and transmission potential, and hence reduce morbidity [2]. However, due to the scarcity of alternative anthelmintics, it is imperative that monitoring systems are designed to detect any change in drug efficacy due to emerging resistance of the parasites against benzimidazoles [3]–[7].

At present, the fecal egg count reduction test (FECRT) is recommended to monitor anthelmintic efficacy against STH in animal [8] and public health [9]. Guidelines on how to conduct a FECRT in public health were published by the World Health Organization (WHO) in the late 1990s [10], providing recommendations on sample size (∼200 infected subjects), stool sampling (two stool samples of two different days both before and after administration of drugs), the detection limit of the method to quantify the number of eggs (Kato-Katz thick smear with a detection limit of 24 eggs per gram of stool (EPG)) and thresholds defining reduced efficacy (FECRT <70% for *A. lumbricoides* and FECRT <50% for *T. trichiura* and hookworm). However, the current guidelines have some important weaknesses. At first, the level of understanding of the effects of the factors inherent both to study design (sample size, stool sampling and the fecal egg count (FEC) method) and host-parasite interactions (level of egg excretion and level aggregation of STH infections across host populations) to support these guidelines is poor. In veterinary sciences, there is empirical evidence that low FEC may thwart interpretation of FECRT results, particularly when sample size is small and/or detection limit of the FEC method is low [11], [12]. As a consequence of this, it is most likely that performing a FECRT across the three STHs, would require a different study design, and this solely due to the differences in fecundity (*A. lumbricoides*≫hookworm>*T. trichiura* [1]). Another important issue of the current guidelines is the additional technical and financial resources that are required to monitor anthelmintic efficacy. Based on the cost assessment of the Kato-Katz thick smear for STH diagnosis by Speich and colleagues in epidemiological surveys in an African setting [13], it can be deduced that the re-examination already would require US$ 3.46 per subject. Therefore, any effort to reduce the cost and the complexity of a surveillance system is desirable. Finally, recent efficacy trials performed in seven countries across Africa, Asia, and Latin America questioned the validity of the thresholds for reduced efficacy [9], as a single dose of albendazole revealed to be highly efficacious against both *A. lumbricoides* (FECRT >99%) and hookworm (FECRT >90%). As a consequence of this, it was proposed to adopt the current thresholds of reduced efficacy to <95% and <90% for *A. lumbricoides* and hookworm, respectively.

The aim of the present study was to assess the impact of sample size, detection limit of the FEC method, level of egg excretion, and aggregation of FEC on the interpretation of the FECRT. To this end, data were generated using a statistical simulation and analyzed using tree based-models. The outcome of these trees was subsequently validated on five efficacy trials previously conducted in Africa, Asia, and Latin America. From the results, we propose cost-effective study designs to successfully monitor anthelmintic drug efficacy in future anthelmintic treatment programs.

## Methods

The study consisted of three consecutive methodological procedures. First, data were generated using a simulation in which the ‘true’ drug efficacy (TDE) was evaluated by the FECRT under varying conditions of sample size, detection limit of the FEC methods, level of excretion and aggregation of FEC across the host population. Subsequently, the obtained data were analyzed using tree-based models, to determine their impact on the interpretation of FECRT and assess critical values in terms of specificity to detect normal efficacy and sensitivity to detect reduced efficacy. Finally, the outcome of these trees was validated on five trials previously conducted to assess the efficacy of a single dose albendazole (400 mg) in school children resident in Africa, Asia, and Latin America.

### Data generation

Data were generated by Monte Carlo simulation as previously described by Dobson et al. (2009) [14] and which was extended by varying sample size, detection limit of the FEC method, level of eggs excreted, and level of aggregation of eggs across the hosts. To fully understand this simulation, the various steps will be explained in more detail.

First, the distribution of parasites within the host population before administration of drugs was defined by a negative binomial distribution. This distribution is determined by two parameters: the mean level of egg excretion across subjects (mean pre-drug administration (pre-DA) FEC) and the level of aggregation of FEC across subjects (k). Low values of k indicate that only few subjects are excreting the majority of eggs, where high values indicate that egg counts are more normally distributed across the host population. From this pre-defined distribution, a number of individual subjects were randomly drawn representing the sample size. An example is given in Table 1, where the outcome of such a random sample is shown for a mean pre-DA FEC = 250 EPG, k = 1 and sample size = 6.

**Table 1:** A detailed example to illustrate the data generated by Monte Carlo simulation.

Host ID	Pre-drug administration FEC (EPG)		Post-drug administration FEC (EPG)	
	*True counts*	*Observed counts*	*True counts*	*Observed counts*
A	796	840	398	288
B	120	168	60	0
C	172	144	86	96
D	212	288	106	120
E	258	240	129	168
F	100	0	50	24
Mean	276.2	280	138.1	116
FECRT (%)			50.0	58.6

The table shows a random sample of six subjects (A–F) drawn from a parasite-host population with a mean pre-drug administration fecal egg count (FEC) of 250 eggs per gram of stool (EPG) and a k-value of 1, for a sample size of six in which a drug with a ‘true’ drug efficacy of 50% was evaluated by the fecal egg count reduction test using a detection technique with a detection limit of 24 EPG.

The pre-DA FEC observed, however, will be different from the ‘true’ pre-DA FEC due to the variation (i.e. stochasticity) introduced by sampling eggs associated with the FEC method. This component of variation was simulated using a Poisson distribution defined by the expected number of eggs counted ( = ‘true’ host FEC/detection limit). In Table 1, the expected number of eggs to be counted when using a FEC method with a detection limit of 24 EPG (*in casu* the standard Kato-Katz thick smear) for subject A with a ‘true’ subject FEC of 796 EPG equaled 33.2 eggs (796/24). A random sample was then drawn from this pre-defined Poisson distribution, and for this sample 35 eggs were observed, which was multiplied by 24 (detection limit) to obtain an observed pre-DA FEC of 840 EPG. This procedure was repeated for each of the six subjects.

In order to simulate a TDE of 50%, the ‘true’ pre-DA FECs were multiplied by 0.5 (1-TDE). The observed FEC after the administration of the drug (post-DA FEC) was generated as described above for the pre-DA FEC. Subsequently, the FECRT was calculated as described in the formula below, resulting in an observed reduction of 58.6% for the example provided in Table 1. It is important to note that only one sample is examined per subject and that all subjects are included in the calculation of the FECRT, even those for whom the observed pre-DA FEC equaled zero. Finally, the entire process was iterated 500 times, to obtain 500 estimates of FECRT for this pre-defined parasite population, sample size, detection limit, and TDE.
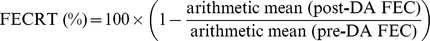



The parasite-host population parameter values chosen for mean pre-DA FEC (50, 100, 150, 200, 250, 500, 750, and 1000 EPG) and k (0.01, 0.025, 0.05, 0.075, 0.1, 0.25, 0.5, 0.75, 1, 1.5, and 2) were based on previously conducted studies where STH were quantified [9], [15], [16]. The values for the sample size were 6, 10, 15, 20, 25, 50, 75, 100, 125, 150, 175, and 200, covering a large range of applied sample sizes to determine drug efficacy against STH [16]. The values for the detection limit represented those of four currently used FEC methods both in human and veterinary parasitology: FLOTAC (detection limit = 1 and 2 EPG) [17], FECPAK (detection limit = 5 and 10 EPG) (http://www.fecpak.com), Kato-Katz thick smear (detection limit = 12 and 24 EPG) [18] and McMaster (detection limit = 25, 33.3, and 50 EPG) [19]. The FEC methods used in veterinary medicine were included in this analysis because they have recently been validated for the diagnosis of STH in public health (FLOTAC [20] and McMaster [21]). In addition, the inclusion of each of these additional assays allowed assessing the impact of detection limit in greater depth. The TDE was set on 50, 60, 70, 80, 82.5, 85, 87.5, 90, 92.5, 95, 97.5, and 99%, resulting in 114,048 combinations (8 (mean pre-DA FEC)×11 (k), 9 (detection limit)×12 (sample size)×12 (TDE)) that were each iterated 500 times.

### Data analysis using tree-based models

The impact of the various factors on the sensitivity and specificity of the FECRT was evaluated. Every TDE that was less than 90 or 95% was considered as a truly reduced efficacy and as truly efficacious if different. Both thresholds have been recently suggested for hookworm and *A. lumbricoides*, respectively [9]. The current threshold for *T. trichiura* (below 50%) was not included, because its remains to be elucidated [22].

A combination of evaluated factors (500 iterations) was considered to be “sensitive” (i.e. true test positive) when a FECRT could be calculated (observed mean pre-DA FEC >0) and a truly reduced efficacy (TDE <90% or <95%) was correctly detected in at least 95% of the iterations or “insensitive” (i.e., false negative) otherwise. A combination of evaluated factors was considered to be “specific” (true test negative) when a FECRT could be calculated (observed mean pre-DA FEC >0) and TDE ≥90 or ≥95% was correctly detected in at least 95% of the iterations or “non-specific” (false positive) otherwise. In the example provided in Table 1, more than 95% of the 500 iterations yielded a FECRT below the defined thresholds, therefore, the FECRT for the combination of a mean pre-DA FEC = 250 EPG, k = 1, detection limit = 24 EPG, and a sample size = 6 was considered ‘sensitive’ to detect the reduced efficacy of 50%. For this combination, the specificity (correctly determine susceptibility when the STH are drug-susceptible) cannot be evaluated as the TDE was below the thresholds for reduced efficacy of both 90 and 95%.

Subsequently, tree-based models (classification trees) were built in R using the packages ‘rpart’ and ‘randomforest” (version 2.10.0, 2009, The R Foundation for Statistical Computing) with both sensitivity and specificity as a binary outcome variable (outcome values are either 0 or 1) and the parasite-host population (mean pre-DA FEC and k), the sample size, the detection limit, and the TDE as continuous predictor variables [23].

### Validation of classification trees

The sample sizes across the different trials were predicted by the classification trees (predicted sample size) and subsequently compared with those estimated by exact inference on the raw data of five previously conducted efficacy trials (required sample size). These trials evaluated the efficacy of a single dose albendazole (400 mg) against *A. lumbricoides* (four out of five trials), *T. trichiura* (three out of five trials) and hookworm infections (all five trials) in school children in three countries in Africa (Cameroon, Ethiopia and Tanzania), one country in Asia (Cambodia) and one Latin American country (Brazil) [9]. These trails were selected for two reasons. First, they were standardized in terms of the follow-up (between 14 and 30 days after the administration of drug), the detection technique (the McMaster egg counting method, detection limit = 50 EPG) and statistical analysis (see formula above). Second, the prevalence of STH before the drug administration exceeded 20% in each of these trials, and hence meeting the criteria to implement preventive chemotherapy programs [24]. For this validation all subjects screened at baseline were included (subjects might be falsely classified as non-infected due to the lack of sensitivity of the McMaster FEC method). However, subjects with a baseline FEC of 0 EPG were not treated nor re-examined at follow-up. To include these subjects it was assumed that the FEC at follow-up of these non-infected subjects (falsely/truly) also equaled zero after drug administration. In addition to this, a number of infected subjects did not provide a stool sample at follow-up. These subjects were replaced by a random sample of subjects for which complete data were available. The sample size, prevalence, mean pre-DA FEC, the aggregation of the FEC (k = (arithmetic mean FEC)^2^/(variance FEC - arithmetic mean FEC)) and the FECRT observed in these trials are summarized in Table 2. For the validation of the statistical methods, these values observed for FECRT, mean pre-DA FEC and k are considered to be ‘true’ values.

**Table 2:** The five trials used to validate the outcome of the classification trees.

Country (number of subjects, prevalence of STH)	Mean pre-DA FEC (EPG)	k	FECRT	Threshold of 90%		Threshold of 95%	
				Required	Predicted	Required	Predicted
***Brazil (n = 350, 30.6%)***							
*A. lumbricoides*	1,353	0.063	100	17	50–200	17	50–200
Hookworm	101	0.097	97.5	**30**	**10–49**	**176**	**50–200**
***Cambodia (n = 1,026, 31.9%)***							
Hookworm	183	0.121	97.6	**20**	**10–49**	**75**	**50–200**
***Cameroon (n = 1,485, 44.1% )***							
*A. lumbricoides*	2,906	0.061	99.2	12	50–200	15	50–200
*T. trichiura*	331	0.042	39.2	32	50–200	22	50–200
Hookworm	59	0.015	93.0	**4405**	**>200**	*>5000*	*50–200*
***Ethiopia (n = 410, 61.0%)***							
*A. lumbricoides*	1,293	0.083	100	7	10–49	7	50–200
*T. trichiura*	110	0.065	92.4	**1152**	**>200**	*1099*	*50–200*
Hookworm	74	0.144	99.7	**12**	**10–49**	14	50–200
***Tanzania (n = 509, 95.3%)***							
*A. lumbricoides*	2,697	0.264	100	3	10–49	4	50–200
*T. trichiura*	832	0.781	52.0	6	10–49	4	50–200
Hookworm	706	0.572	95.3	*78*	*10–49*	**>5000**	**>200**

Cases highlighted in bold indicate an agreement between the required and the predicted sample size (the required sample size fell within the sample size interval predicted). Cases highlighted in italic indicate an underestimation of the required sample size (required sample size > upper limit of the predicted sample size interval). In the remaining cases, the required sample size was overestimated (required sample size > lower limit of the predicted sample size).

The overall protocol of this multi-country study was approved by the ethics committee of the Faculty of Medicine, Ghent University (no. B67020084254) and was followed by a separate local ethical approval for each study site. For Brazil, approval was obtained from the institutional review board (IBR) from Centro de Pesquisas René Rachou (no. 21/2008), for Cambodia from the national ethic commitee for health research, for Cameroon from the national ethics committee (no. 072/CNE/DNM08), for Ethiopia from the ethical review board of Jimma University, for India from the IBR of the Christian Medical College (no. 6541), for Tanzania (no. 20) from the Zanzibar Health Research Council and the Ministry of Health and Social Welfare, for Vietnam by the Ministry of Health of Vietnam. An informed consent form was signed by the parents of all subjects included in the trials. This clinical trial is registered under the ClinicalTrials.gov, identifier NCT01087099.

**Figure 1:**
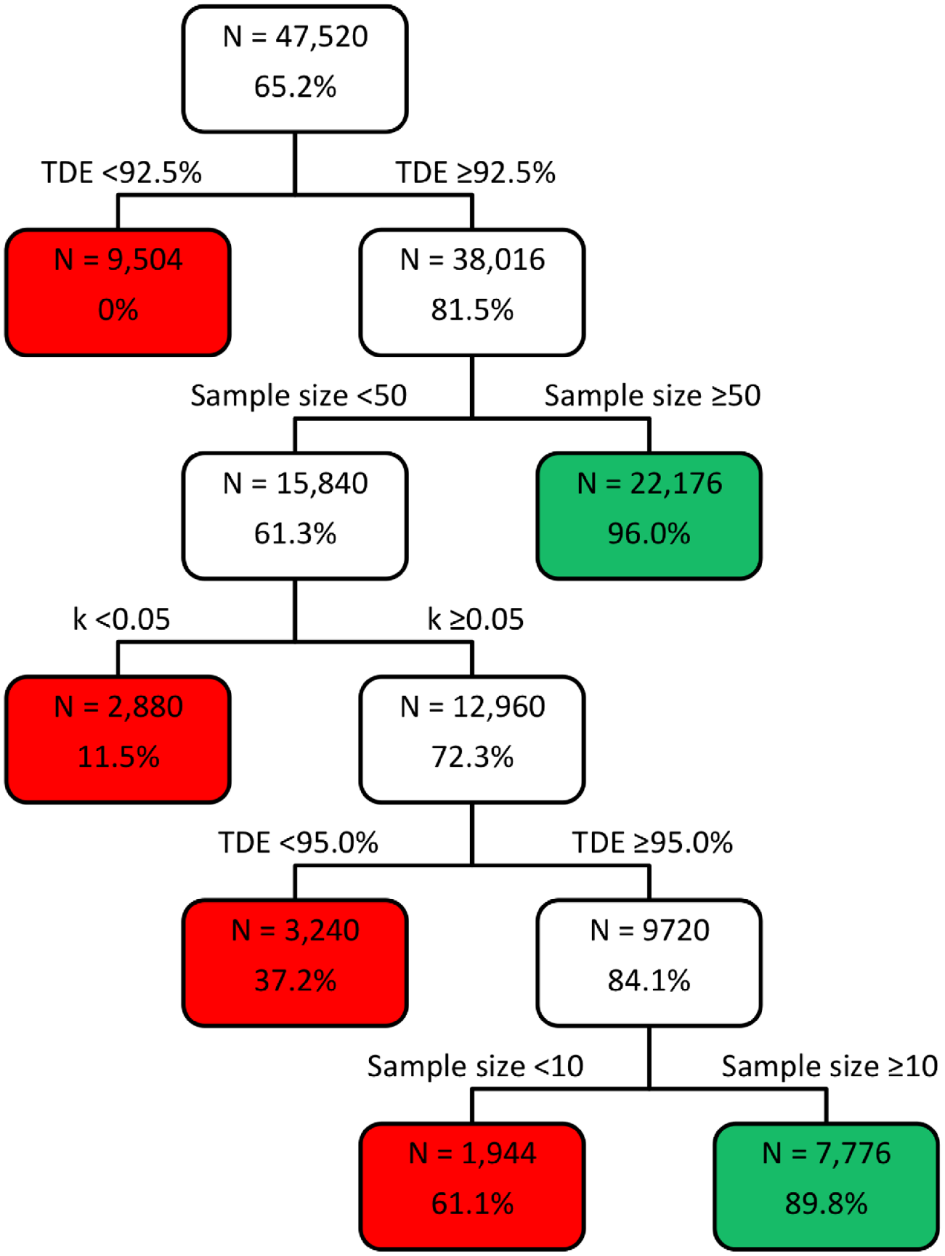
The classification tree of the factors affecting FECRT specificity (TDE ≥90%). The classification tree of the factors affecting FECRT specificity (%) (correct detection of a ‘true’ drug efficacy (TDE) ≥90%); factors included mean fecal egg count (FEC) before administration of drugs (pre-DA FEC), aggregation of FEC (k), sample size, detection limit, and TDE. N = number of combinations.

**Figure 2:**
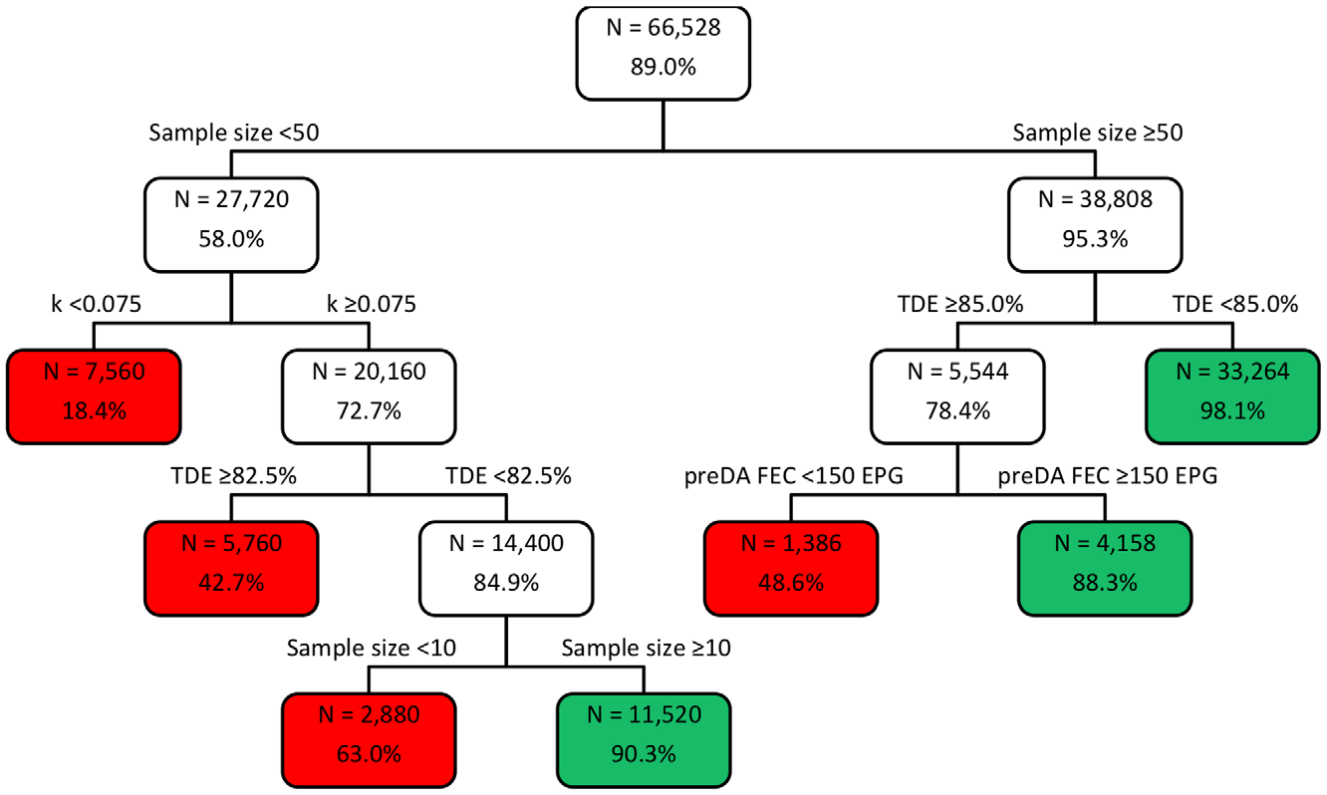
The classification tree of the factors affecting FECRT sensitivity (TDE <90%). The classification tree of the factors affecting FECRT sensitivity (%) (correct detection of a reduced efficacy when ‘true’ drug efficacy (TDE) <90%); factors included mean fecal egg count (FEC) before administration of drugs (pre-DA FEC), aggregation of FEC (k), sample size, detection limit, and TDE. N = number of combinations.

**Figure 3:**
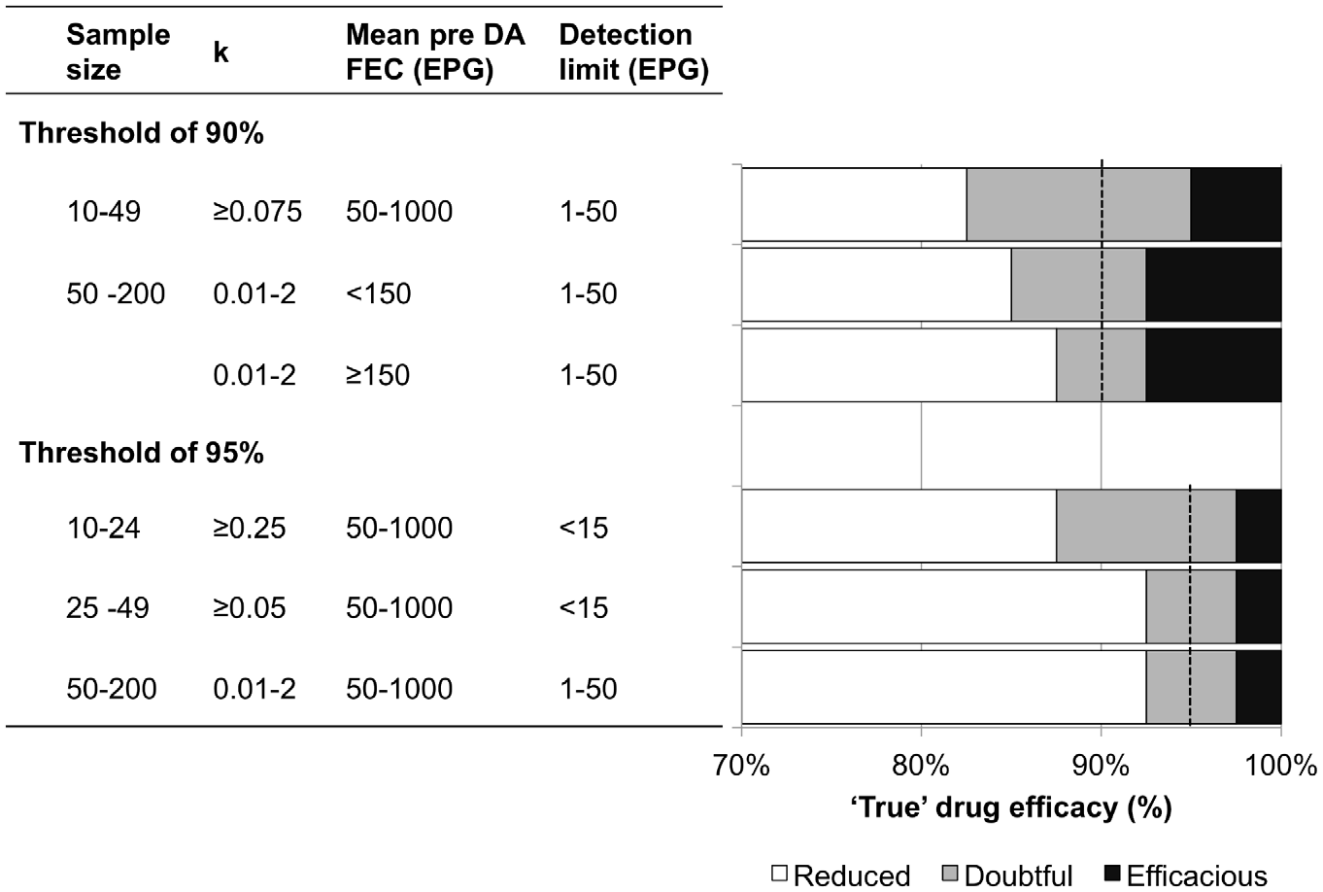
The detection of reduced efficacy when defined as ‘true’ drug efficacy <90% and <95%. Only combinations of sample size (n), mean fecal egg count before drug administration (mean pre-DA FEC), detection limit and aggregation (k), which resulted in a reliable FECRT (sensitivity (Se) and specificity (Sp) ≥85%) are shown. Also shown are TDE limits for Se and Sp, which caused Se and Sp <85%.

The predicted sample sizes were deduced from the results of the classification trees (Figures 1, 2, S2 and S3) and are shown in Figure 3. For example, the predicted sample size to correctly diagnosis a reduced efficacy against *A. lumbricoides* in the Brazilian trial (FECRT = 100%, mean pre-DA FEC = 1,353 EPG and k = 0.063) ranged from 50 to 200 for both thresholds defining reduced efficacy. When none of the combinations resulted in a reliable diagnosis, the predicted sample size was set at >200, as this was the largest sample size examined in the classification trees. This was for example the case for the efficacy against *T. trichiura* (FECRT = 92.4%, mean pre-DA FEC = 110 EPG and k = 0.065) in the Ethiopian trial, and this for both thresholds.

The required sample size based on the raw data of the different trials was estimated by bootstrap analysis (re-sampling with replacement and 10,000 iterations), as at present no formulae are available to calculate sample size for the correct diagnosis of reduced efficacy. In this analysis, different sample sizes were analyzed in order to determine the smallest sample size for which FECRT could be calculated (mean pre-DA FEC >0) and a truly reduced efficacy (TDE <90% or <95%) was correctly detected in at least 95% of the iterations or when a TDE ≥90 or ≥95% was correctly detected in at least 95% of the iterations. The outcome of the bootstrap analysis for Brazilian trial against *A. lumbricoides* described above is illustrated in Figure S1. As the sample size increase, the probability of correctly detecting a reduced efficacy increased. The required sample size based on this trial was 17 for both thresholds.

In 24 cases the agreement between the required and the predicted sample size was assessed (two thresholds (90 and 95%)×12 FECRT (four for *A. lumbricoides*, three for *T. trichiura* and five for hookworm). There was an agreement between the required and the predicted sample size, if the exact required sample size fell within the predicted sample size interval. For cases where the required sample size did not fall within the predicted sample size interval, it was assessed whether the required sample size was overestimated (required sample size < lower limit of the predicted sample size interval) or underestimated (required sample size > upper limit of the predicted sample size).

## Results

### Detection of a normal or reduced efficacy for a threshold of 90%

The classification-trees for the specificity to detect efficacy ≥90% and sensitivity to detect reduced efficacy <90% are provided in Figure 1 and 2, respectively. The terminal nodes are colored green when the specificity/sensitivity was reliable (≥85%), and red if different.

The specificity was evaluated in the 47,520 combinations were the TDE was ≥90% and was affected with decreasing importance (increasing number of bifurcations from the root node) by TDE, sample size and aggregation of the FEC (k). The detection limit and mean pre-DA FEC did not considerably influence the specificity, since these parameters did not result in any bifurcation across the classification tree. From the red-green color code to define a reliable specificity, it can be deducted that false positive conclusions concerning reduced efficacy were drawn when the TDE was between 90 and 92.5% (specificity = 0%, n = 9,504). For a TDE ≥92.5%, reliable specificity results depended on the sample size. For small sample sizes (<50 subjects), reliable conclusions could only be drawn when lowly aggregated FEC (k≥0.05) were combined with TDE ≥95% and a sample size of at least 10 subjects (specificity = 89.8%, n = 7,776). For large sample sizes (≥50), specificity was always high, regardless of the aggregation of the FEC and TDE (specificity = 96.0%, n = 22,176).

The sensitivity was evaluated in the remaining 66,528 combinations where the TDE did not exceed 90%. The most important factor affecting the sensitivity was the sample size, followed by both TDE and aggregation of the FEC and finally the mean pre-DA FEC. The detection limit did not considerably influence the sensitivity. For sample sizes <50, reduced efficacies were only correctly diagnosed when lowly aggregated FEC (k≥0.075) were combined with a TDE <82.5% and a sample size of ≥10 (sensitivity = 90.3%, n = 11,520).

For sample sizes ≥50, the diagnosis of reduced efficacy depended on the TDE. For TDE between 85.0% and 87.5%, satisfactory sensitive results were only found when mean pre-DA FEC were high (≥150 EPG) (sensitivity = 88.3%, n = 4,158). For TDE <85.0%, sensitivity was high ( = 98.1, n = 33, 264), regardless of the mean pre-DA FEC.

The combinations that result in a reliable detection of a normal or reduced efficacy (sensitivity and specificity >85%) and their respective TDE limits for which the FECRT cannot reliably provide a correct diagnosis (‘grey’ zone) are summarized in Figure 3. All combinations resulted in a reliable classification of efficacy status, except for small sample sizes (<10) and moderate sample sizes (10–49) combined with highly aggregated FEC (k <0.075). The TDE limits for sensitivity (TDE = 82.5%) and specificity (TDE = 95%) that were least discriminatory occurred for moderate sample sizes (10–49) combined with low aggregated FEC (k≥0.075). Best discrimination TDE limits for sensitivity and specificity were 87.5% and 92.5%, respectively, and occurred for large sample sizes (50–200) combined with high mean pre-DA FEC (≥150 EPG).

### Detection of a normal or reduced efficacy for a threshold of 95%

For the reduced efficacy threshold of 95%, only the combinations, which result in a reliable classification of efficacy status and their TDE limits in which FECRT results are unreliable, are reported (Figure 3). The classification trees of the specificity and sensitivity are provided in Figures S2 and S3, respectively.

Compared to a reduced efficacy defined as TDE <90%, there were three important differences. First, the pre-DA FEC did not affect the diagnosis of reduced efficacy. Second, the detection limit had a considerable impact on the interpretation of FECRT. A detection limit ≥15 did not always allow a reliable FECRT, particularly for sample sizes <50 subjects. Finally, there was a difference in the critical value(s) for sample size (10 and 50 for 90% threshold *vs.* 10, 25, and 50 for the 95% threshold) and aggregation of the FEC (0.075 *vs.* 0.05 and 0.25). The least discriminatory TDE limits for which conclusions were doubtful was found when moderate sample sizes (10–24) were combined with a high detection limit (<15 EPG) and lowly aggregated FEC ≥0.25. For these combinations TDE limits were 87.5% to 97.5% for sensitivity and specificity, respectively. Best discrimination (TDE 92.5% and 97.5% for sensitivity and specificity respectively) was observed for moderate sample sizes (25–49) combined with high detection limits (<15 EPG) and for large sample sizes (≥50 subjects) regardless of the detection limit.

### Validation of the classification trees

The predicted and the required sample sizes across the different trials for both a normal and a reduced efficacy <90 and <95% are provided in Table 2. Overall, there was an agreement between the predicted and the required sample size in eight out of 24 cases (highlighted in bold). In the 16 remaining cases, the required sample size fell out of the interval of predicted sample size. Yet, the required sample was only underestimated in three cases (highlighted in italic). In the remaining 13 cases, the required sample size was overestimated (not highlighted). There was a slight variation in agreement between the required and predicted sample size across the two thresholds defining reduced efficacy. For a threshold of 90%, there was an agreement between the required and the predicted sample size in five cases, whereas this was only observed in three cases for a threshold of 95%. Moreover, two out of the three cases for which the required sample size was underestimated were found for the latter threshold.

## Discussion

In the present study, the most applied test to evaluate anthelmintic drug efficacy against *A. lumbricoides* and hookworm in public health, was virtually performed under varying conditions of sample size, detection limit of the FEC method, level of excretion, and aggregation of eggs within the host population. Subsequently, tree-based models were built to assess the impact of these factors on the specificity and the sensitivity to detect normal or reduced efficacy. Finally, the outcomes of these models were validated on different efficacy trials done in Africa, Asia and Latin America.

The present study provides novel insights into three aspects of FECRT. The first important finding is that a successful interpretation of the FECRT is not always possible and that this is not always due to factors inherent to the design of a study, but can also be caused by factors inherent to host-parasite interactions (e.g., level of excretion and aggregation of eggs within the host population). For a threshold of 90% (hookworm), unreliable FECRT results were obtained when sample sizes were small or when moderate sample sizes were combined with highly aggregated FEC. For a threshold of 95% (*A. lumbricoides*), diagnostic performance was poor when sample sizes are small and when moderate sample sizes were combined with highly aggregated FEC and/or with FEC methods with a low detection. Second, our results highlight that the interval of TDE for which the FECRT remains inconclusive (so called ‘grey’ zone, Figure 3) is unexpectedly small, ranging from 87.5% to 92.5% for hookworm and from 92.5% to 97.5% for *A. lumbricoides*. Third, the study design with the greatest discriminatory power to classify drug efficacy requires examination of 50 to 200 subjects, for both hookworm and *A. lumbricoides*. For this interval of sample sizes, there were no additional requirements on the detection limit of the FEC method and the level of aggregation of the FEC did considerably influence the interpretation of the FECRT. Only for hookworm, mean pre-DA FEC <150 EPG led to a less reliable interpretation of FECRT, as the ‘grey’ zone ranged from 85.0% to 92.5%.

Overall, our findings contrast sharply with the recommendations provided by WHO [10], explained by two main reasons. First, our analysis indicates that including subjects with any STH status (absence (true or falsely) or presence of eggs in stool) will not affect the final interpretation of the FECRT, yet this allows a dramatic reduction in the required sample size. For example, monitoring drug efficacy in a low risk-population (STH prevalence = 20%) would require screening 1,000 subjects (in order to obtain a sample size of 200 infected subjects) according to WHO guidelines, whereas according to our findings only 50 to 200 subjects are required. This, however, remains a large interval of possible sample sizes, which requires further refinement. The outcome of the efficacy used to validate the tree-based models, indicated that a minimum of 200 subjects are recommend, as this sample size allowed for a reliable detection of normal or reduced efficacy in all 14 trials where the FECRT fell outside the ‘grey’ zone. Second, our results do not support the need for four fecal samples per subject (two before and two after administration of drugs), and hence will further reduce the costs to implement a monitoring system. This is mainly based on the fact that in the present simulation of FECRT based on two stool samples per subject (one before and one after administration of drugs), and hence partially ignoring any variation in FEC due to differences in FEC across days, did not result in an underestimation of the required sample size. Moreover, the detection limit of the FEC method revealed to be less critical than anticipated, highlighting the importance of the feasibility of the FEC method used. Recently, Kato-Katz thick smear, FLOTAC, and McMaster egg counting methods have been compared for their feasibility in diagnosing STH [13], [25]. Of these three methods, McMaster egg counting method was considered the most feasible, as the procedure does not include centrifugation steps (*vs.* FLOTAC) and allows quantifying all STH in one single reading (*vs.* Kato-Katz thick smear). Based on these studies assessing the cost of these diagnostic methods, it is estimated that the average time for preparing, reading and examining one stool sample is roughly 5 min for McMaster egg counting method, 10 min for Kato-Katz thick smear, and 26 min for FLOTAC [13], [25].

The combination of different statistical procedures (Monte Carlo simulation and tree-based models), allowed for a cost-reduced data generation providing a decision support framework rather than a descriptive analysis. At present, both approaches are increasingly applied in various aspects of both public [26] and animal health [27]. However, this statistical approach to evaluate FECRT has limitations that must be acknowledged. First, it is assumed that worm abundance is adequately reflected by FECs, yet it remains unclear whether this holds true for STH infections in humans, particularly for hookworms. For this STH, a density dependent fecundity - female worms that survived the anthelmintic treatment produce relatively more eggs - has been described in dogs (*Ancylostoma caninum*) [28]. These density dependent effects imply a reduced drug efficacy for subjects with higher pre-intervention FEC, but this has not yet been observed in human trials [9]. Secondly, the generation of the observed FEC did not consider additional variation caused by properties of the detection technique beyond the detection limit, which impedes a straightforward extrapolation of the findings across FEC methods. Both the specific density of the flotation solution (large difference in mass of parasite eggs) [29] and the inclusion of a centrifugation step (increasing FEC when included) have an important impact on the FEC obtained by various FEC methods [30]. For Kato-Katz thick-smear, the templates used to substitute the calibrated weight of examined stool by a calibrated volume introduce an additional variation [25], [31]. Additionally, differences in processing samples across investigators or laboratories should not be neglected [25], [31]–[33]. As a consequence, it will become necessary to quantitatively validate the ability of both old and novel techniques to determine true FECRT rather than simply compare their ability to correctly diagnose the presence or absence of infections [12]. Thirdly, this simulation did not include any STH populations defined by a mean baseline FEC <50 EPG and/or a k <0.01. Although this kind of populations are to be expected after a successful implementation of preventive chemotherapy programs, the simulation still represents a significant part of the populations at risk of STH infections. This is in particular when the target to administer anthelmintics to at least 75% of the population at risk by 2010 set by World Health Assembly Resolution 54.19 in 2001, was not met (coverage was <20% in 2008) [34]. Moreover, it is most likely that by then the endpoints of these programs will shift from ‘reducing morbidity’ to ‘eradicating’ of STH infections, which will demand a shift in study design and efficacy indicators of monitoring programs of anthelmintic efficacy.

In conclusion, this study points out that the final interpretation of the FECRT was affected by a complex interplay of factors inherent to both study design and host-parasite interaction. The results also indicate that current WHO guidelines need to be revised. Based on the current study and the outcome of previously assessed efficacy trials [9], we propose to include a minimum of 200 subjects independent of STH status (subjects who are not excreting eggs can also be included) and to examine two stool samples per subject (one at baseline and one at follow-up). In this set-up, the choice of FEC method is not critical and arithmetic-mean based FECR <95% for *A. lumbricoides* and <90% for hookworms can be used as indicators for reduced efficacy and potential presence of drug resistance against albendazole.

## Supporting Information

Figure S1 **The required sample size based on bootstrap analysis for the Brazilian trial against** ***A. lumbricoides*** **.** The required sample size based on bootstrap analysis (10,000 iterations) for the correct diagnosis of reduced efficacy <90% and <95% in the Brazilian trial against *A. lumbricoides*.
(TIF)

Figure S2 **The classification tree of the factors affecting FECRT specificity (TDE ≥95%).** The classification tree of the factors affecting FECRT specificity (%) (correct detection of a ‘true’ drug efficacy (TDE) ≥95%); factors included mean fecal egg count before administration of drugs (pre-DA FEC), aggregation of FEC (k), sample size, detection limit, and TDE. N = number of combinations.
(TIF)

Figure S3 **The classification tree of the factors affecting FECRT sensitivity (TDE <95%).** The classification tree of the factors affecting FECRT sensitivity (%) (correct detection of a reduced efficacy when ‘true’ drug efficacy (TDE) was <95%); factors included mean fecal egg count before administration of drugs (pre-DA FEC), aggregation of FEC (k), sample size, detection limit, and TDE. N = number of combinations.
(TIF)
