# Supplementary figures and images for: Novel Insights in the Fecal Egg Count Reduction Test for Monitoring Drug Efficacy against Soil-Transmitted Helminths in Large-Scale Treatment Programs
Source: PLoS Negl Trop Dis. 2011 Dec 13;5(12):e1427. doi: 10.1371/journal.pntd.0001427 (PMC3236725; doi:10.1371/journal.pntd.0001427)

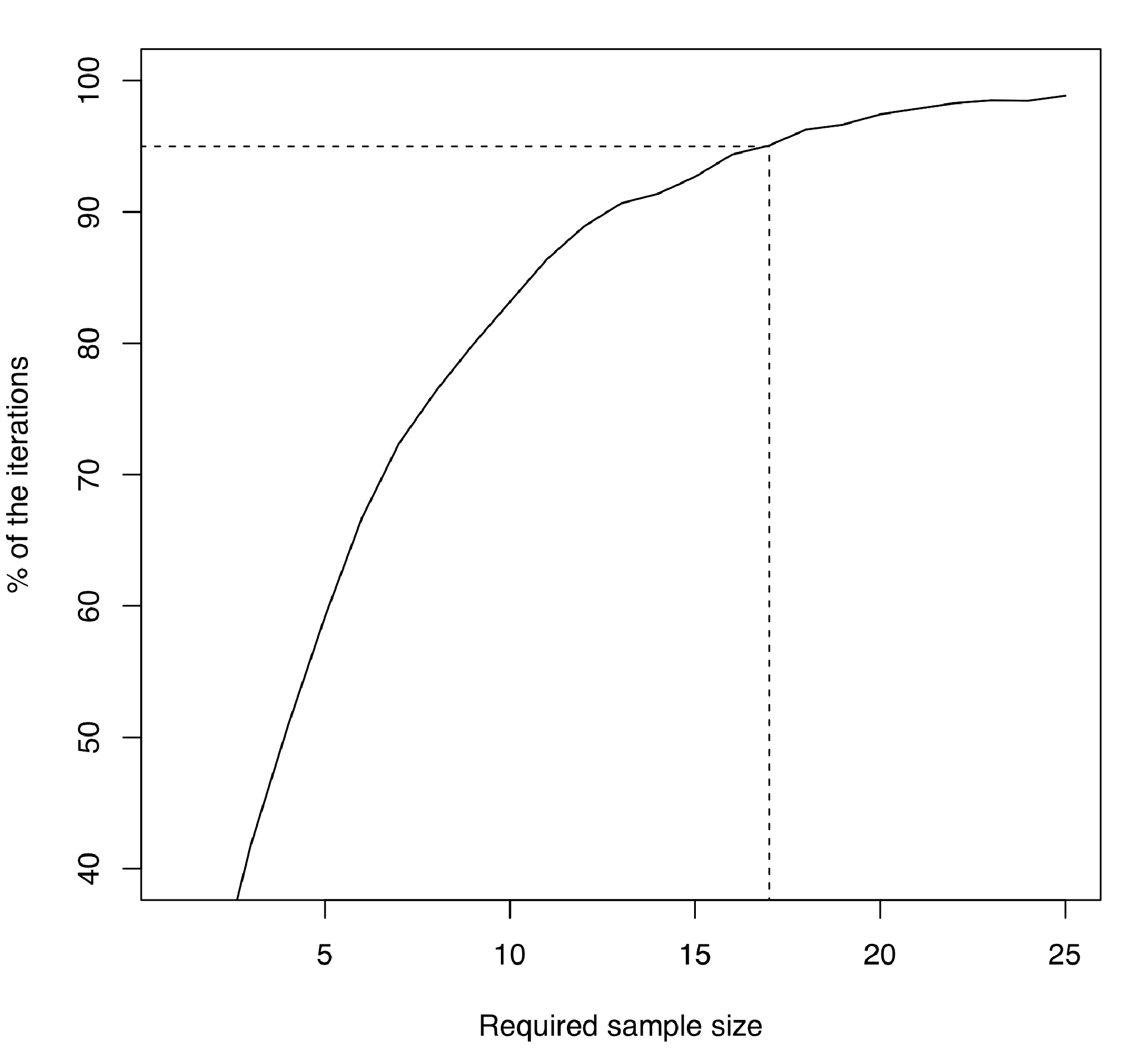

Supplement: Figure S1 — The required sample size based on bootstrap analysis for the Brazilian trial against A. lumbricoides . The required sample size based on bootstrap analysis (10,000 iterations) for the correct diagnosis of reduced efficacy <90% and <95% in the Brazilian trial against A. lumbricoides. (TIF) [file pntd.0001427.s001.tif]

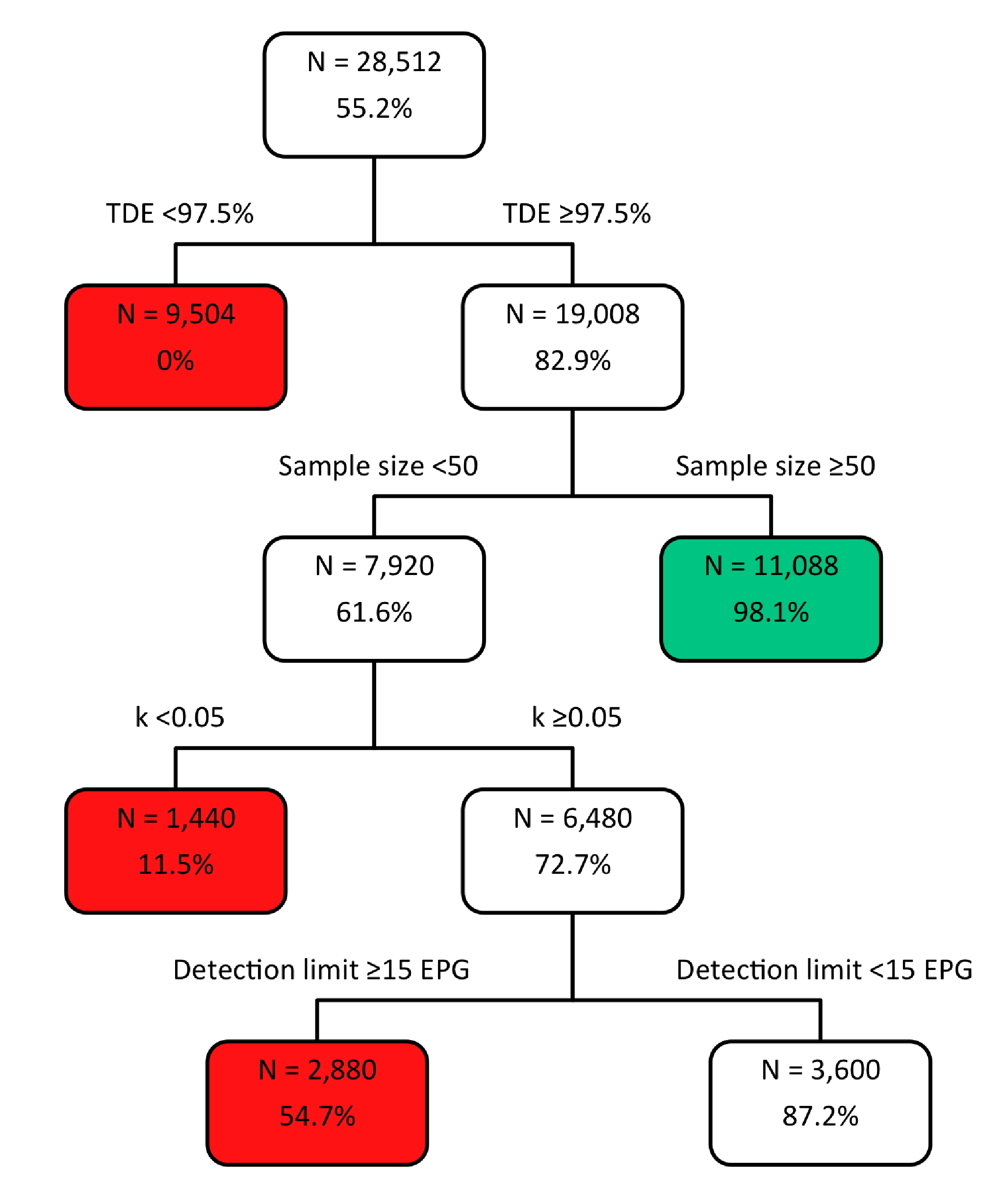

Supplement: Figure S2 — The classification tree of the factors affecting FECRT specificity (TDE ≥95%). The classification tree of the factors affecting FECRT specificity (%) (correct detection of a ‘true’ drug efficacy (TDE) ≥95%); factors included mean fecal egg count before administration of drugs (pre-DA FEC), aggregation of FEC (k), sample size, detection limit, and TDE. N = number of combinations. (TIF) [file pntd.0001427.s002.tif]

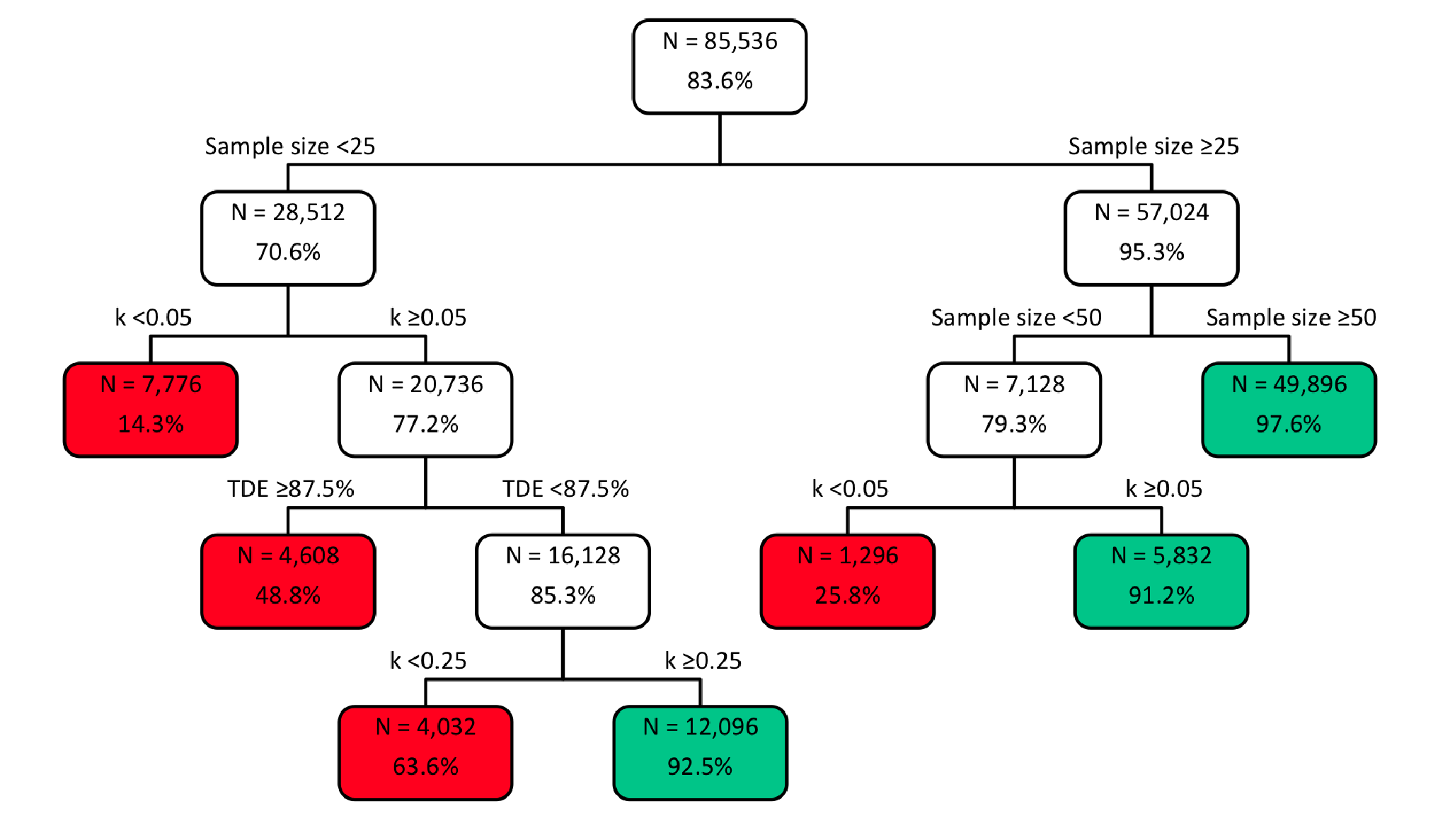

Supplement: Figure S3 — The classification tree of the factors affecting FECRT sensitivity (TDE <95%). The classification tree of the factors affecting FECRT sensitivity (%) (correct detection of a reduced efficacy when ‘true’ drug efficacy (TDE) was <95%); factors included mean fecal egg count before administration of drugs (pre-DA FEC), aggregation of FEC (k), sample size, detection limit, and TDE. N = number of combinations. (TIF) [file pntd.0001427.s003.tif]
